# Explaining recovery from coma with multimodal neuroimaging

**DOI:** 10.1007/s00415-024-12591-y

**Published:** 2024-08-01

**Authors:** Polona Pozeg, Jane Jöhr, John O. Prior, Karin Diserens, Vincent Dunet

**Affiliations:** 1 Departement of Medical Radiology, Lausanne University Hospital and University of Lausanne, Rue du Bugnon 46, 1011 Lausanne, Switzerland; 2 Acute Neurorehabilitation Unit, Department of Clinical Neurosciences, Lausanne University Hospital and University of Lausanne, 1011 Lausanne, Switzerland; 3 Department of Nuclear Medicine and Molecular Imaging, Lausanne University Hospital and University of Lausanne, 1011 Lausanne, Switzerland

**Keywords:** Disorders of consciousness, DWI, fMRI, PET, Brain injury, Recovery

## Abstract

The aim of this prospective, observational cohort study was to investigate and assess diverse neuroimaging biomarkers to predict patients’ neurological recovery after coma. 32 patients (18–76 years, *M* = 44.8, SD = 17.7) with disorders of consciousness participated in the study. Multimodal neuroimaging data acquired during the patient’s hospitalization were used to derive cortical glucose metabolism (^18^F-fluorodeoxyglucose positron emission tomography/computed tomography), and structural (diffusion-weighted imaging) and functional connectivity (resting-state functional MRI) indices. The recovery outcome was defined as a continuous composite score constructed from a multivariate neurobehavioral recovery assessment administered upon the discharge from the hospital. Fractional anisotropy-based white matter integrity in the anterior forebrain mesocircuit (*r* = 0.72, *p* < .001, 95% CI: 0.87, 0.45), and the functional connectivity between the antagonistic default mode and dorsal attention resting-state networks (*r* = − 0.74, *p* < 0.001, 95% CI: − 0.46, − 0.88) strongly correlated with the recovery outcome. The association between the posterior glucose metabolism and the recovery outcome was moderate (*r* = 0.38, *p* = 0.040, 95% CI: 0.66, 0.02). Structural (*adjusted R*^2^ = 0.84, *p* = 0.003) or functional connectivity biomarker (*adjusted R*^2^ = 0.85, *p* = 0.001), but not their combination, significantly improved the model fit to predict the recovery compared solely to bedside neurobehavioral evaluation (*adjusted R*^2^ = 0.75). The present study elucidates an important role of specific MRI-derived structural and functional connectivity biomarkers in diagnosis and prognosis of recovery after coma and has implications for clinical care of patients with severe brain injury.

**Supplementary Information:**

The online version contains supplementary material available at 10.1007/s00415-024-12591-y.

## Introduction

Coma is a state of prolonged unarousable unresponsiveness following severe brain injury. Recovery occurs in a gradual but not necessarily definite restoration of arousal, awareness, and responsiveness [1]. Bedside clinical evaluation using neurobehavioral scores, such as the Glasgow Coma Scale [2] or the Coma Recovery Scale-Revised (CRS-R) [3], remains the standard approach to assess the level of impaired consciousness and predict the outcome [4]. Clinical evaluation is indispensable for establishing the proper diagnosis and treatment plan for the patient’s care; however, accurate detection of subtle signs of conscious awareness may often be hindered. The misdiagnosis rate following bedside examination can reach 40% [5, 6], influenced by biases, such as the examiner, the environment, and/or the patient [7, 8]. In the latter, sensory impairments or neurological conditions affecting motor functions, language and praxia may conceal the patient’s ability to interact with the environment despite being conscious and mimic disturbances of consciousness [9].

Complementing clinical examination with neuroimaging can significantly improve the patient’s diagnosis, prognosis, and subsequently their treatment plan. Owen’s et al. [10] seminal study demonstrated the successful use of an active imagery task paradigm during functional MRI (fMRI) to identify covert awareness in a patient behaviorally diagnosed being in a vegetative state. This paradigm has been since applied to larger cohorts of patients, confirming the presence of covert awareness in a proportion of unresponsive patients [11–15]. These patients show the ability to willfully modulate their brain activity following a command by engaging in motor or spatial imagery. Due to a clear dissociation between their motor output and residual cognitive abilities, their condition has been defined as a cognitive motor dissociation (CMD) [16]. While the neuromodulation task showed very good sensitivity in healthy subjects [17], assessing its detection accuracy in behaviorally unresponsive patients is impossible due to the absence of an independent, “ground-truth” measure of awareness, other than behavior [18]. It, therefore, represents a great risk for false negatives since a severe brain injury often gravely impacts functioning in multiple cognitive domains required to perform the fMRI mental imagery task [19].

Apart from the above-mentioned task-fMRI, the application of neuroimaging techniques to improve clinical diagnosis and predict recovery has been intensely studied, using diverse imaging methods, for example, structural imaging to gain qualitative and quantitative information about structural damage, or functional imaging using varying passive task paradigms and task-free methods [20] offering an insight into brain activity. Structural connectivity focused analyses based on microstructure diffusion-weighted imaging (DWI), revealed the significance of specific white matter track integrity in predicting the recovery from coma [21–23]. The anterior forebrain mesocircuit has been suggested as a prominent model to explain the common underlying mechanism for disorders of consciousness of different etiologies [24, 25]. The main components of the circuit: medial frontal and anterior cingulate cortex, central thalamus and the striatum, form a supporting architecture for brain arousal regulation of excitatory input from the brainstem [25]. The level of preserved integrity of the mesocircuit structures and their structural connectivity showed to be correlated with the degree of recovery after coma [23, 26–30].

The anterior forebrain mesocircuit also plays an important interactive role in sustaining and moderating neural activity of the cortical fronto-parietal networks [25, 31]. On the one hand, these networks consist of the default mode network (DMN; the medial prefrontal cortex, the posterior cingulate cortex, precuneus and the angular gyri), which is activated during passive rest conditions, internally oriented attention, and during self-referential processes [32]. On the other hand, the DMN is inhibited during tasks that require externally oriented attention, and activate lateral fronto-parietal and inferior parietal regions [33]. The strongest anti-correlation has been observed with the dorsal attention network (DAN), principally composed of the frontal eye fields and intraparietal sulcus [34, 35]. The anti-correlation between the DMN and DAN is an inherent robust feature of the functional organization of the brain and it underlies a segregation of competitive internal and external cognitive mechanisms [36]. Adequate segregation is thought to reflect the brain’s ability to adapt to a changing surrounding by flexibly allocating attention resources and is an indicator of a healthy neural connectivity [36–38]. Accumulating evidence has shown that the within- and between-network connectivity of the DMN and extrinsic networks assessed during resting-state fMRI (rs-fMRI) is attenuated in patients with less favorable outcome after severe brain injury [39–42], possibly being a promising neuroimaging biomarker to assess residual brain function. Similarly, the studies on brain metabolism using the ^18^F-FDG PET/CT showed reduced glucose metabolism in severe brain injury patients with less favorable diagnosis [12, 30], particularly in the posterior cingulate and precuneus [43, 44], which are the central nodes of the DMN [45], as well as the highest interconnected hub in the brain [46].

Despite the accumulating knowledge on neural mechanisms of recovery after brain injury and prominent advances in neuroimaging, determining an accurate prognosis in severe brain injury still remains a difficult challenge with critical consequences for the patient. The use of neuroimaging, while valuable, poses non-negligible cost and accessibility issues. It is hence imperative to assess the value of various neuroimaging biomarkers to optimize the use of resources and improve the prediction of coma outcome. Nonetheless, studies using multimodal neuroimaging measures and comparing their role in the prognosis of recovery in the disorders of consciousness are sparse [30, 39, 47, 48]. Therefore, the goal of this study was twofold. We first evaluated univariate associations between the recovery level and multimodal neuroimaging biomarkers, derived from the DWI, rs-fMRI and ^18^F-FDG PET/CT imaging. In particular, we assessed the mesocircuit structural connectivity and fronto-parietal functional integrity as potential predictors of the recovery after coma. We then compared which combination of the neuroimaging biomarkers can best improve the prediction of the recovery at the post-acute phase with regards to the clinical assessment only. The results of the present study showed that complementing bedside neurobehavioral evaluation with a selective neuroimaging biomarker importantly improves the prediction of recovery after severe brain injury.

## Materials and methods

### Subjects

**Fig. 1:**
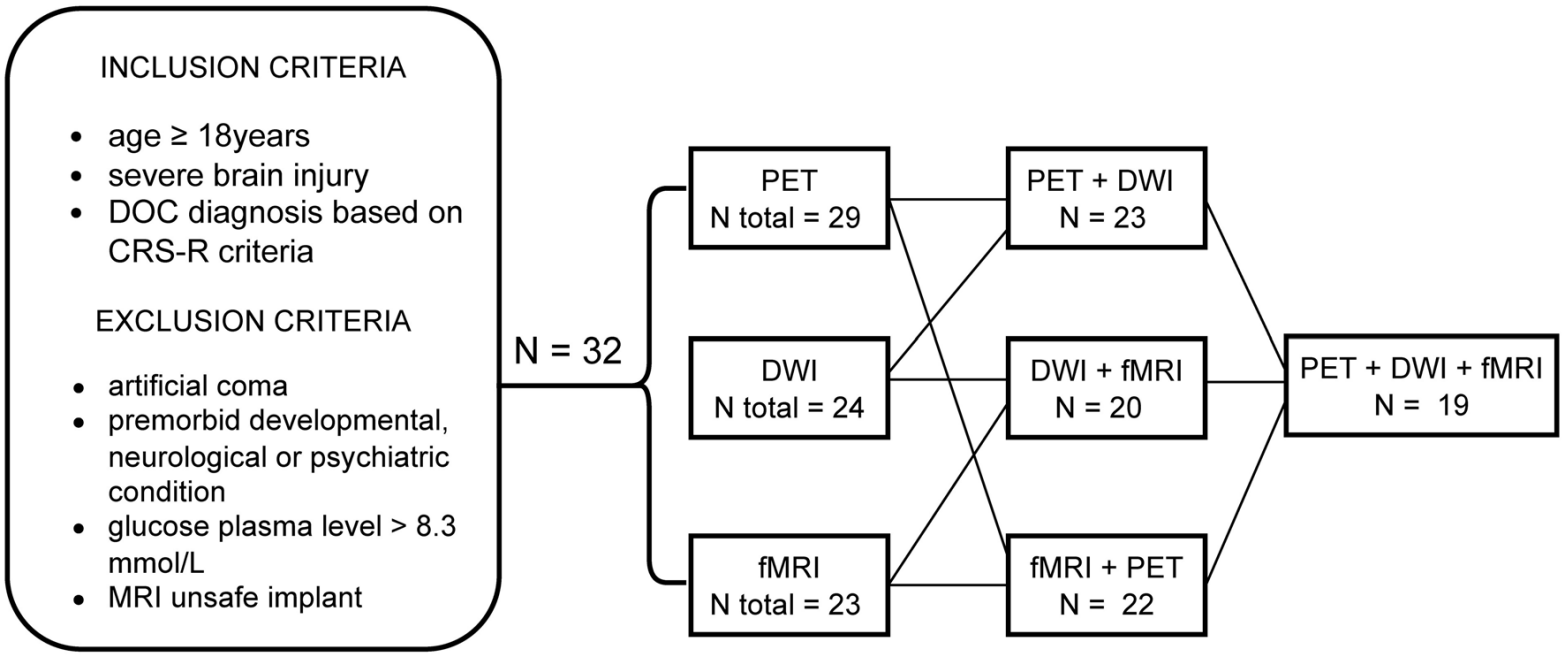
Inclusion flow diagram and the number of patients with imaging data. In total 32 patients were recruited, of which 29 had a ^18^F-FDG PET/CT scan, 24 had a diffusion MRI scan and 23 had a resting-state functional MRI (rs-fMRI). 19 patients had data of all three imaging modalities

Adult patients (≥ 18 years old) admitted to the Acute Neurorehabilitation Unit at the Lausanne University Hospital between the May 1, 2020 and the April 30, 2023 were enrolled in this prospective study. Inclusion criteria were a severe brain injury due to trauma or disease, and a behavioral phenotype of disorders of consciousness based on the clinical consensus for DOC diagnosis, i.e., Coma Recovery Scale—Revised (CRS-R) criteria for coma, unresponsive wakefulness syndrome, or minimally conscious state [3, 49]. Exclusion criteria were artificial coma, premorbid history of developmental, psychiatric or neurological illness resulting in documented functional disabilities at the time of the accident, glucose plasma level > 8.3 mmol/L, an MRI-unsafe device or metal fragment implant (Fig. 1). Only patients with informed consent to participate in the study, obtained from their legal representatives, were included in the study. The study was conducted in compliance with the ethical standards of the Declaration of Helsinki and was approved by the local ethical committee (CER-VD, 142/09).

### Clinical evaluations

The patients’ levels of motor, cognitive, and functional recovery were repeatedly assessed with a set of neurobehavioral evaluation tools during their stay in the unit by an experienced neuropsychologist or neurologist. The patients’ evolution was continuously monitored with the CRS-R first prior to their admission to the unit and followed-up every 7 days during their stay at the unit until the recovery of consciousness according to the CRS-R criteria (i.e., functional use of objects and/or functional communication). The first CRS-R evaluation was complemented with the Motor Behavior Tool—revised [50, 51] to detect subtle signs of motor behavior that could indicate a clinical CMD (cCMD) [52]. Patients with cCMD present subtle signs of conscious perception not accounted for by the CRS-R, and in the absence of decortication/decerebration signs reflect bilateral pyramidal pathway lesion.

A multivariate assessment of patient’s recovery was performed at the discharge from the unit using the Disability Rating Scale (DRS) [53], Rancho Los Amigos Levels of Cognitive Functioning Scale (RLAS) [54], and Functional Ambulation Category (FAC) [55].

### ^18^F-FDG PET/CT and MR imaging acquisition

In line with the study protocol, each patient underwent two scanning sessions 2 weeks apart. Each session consisted of first ^18^F-FDG PET/CT scan followed with an MRI scan the following day. The analyses were performed on the neuroimaging data of the first session; however, in the case of missing or insufficient quality data, we used the neuroimaging data of the second session.

MR data were collected on a 3T Siemens Skyra fit scanner (*n* = 14) and 3T Siemens Magnetom Vida scanner (*n* = 16; Erlangen, Germany) using the same scanning protocol.

Anatomic T1-weighted 3D magnetization-prepared rapid acquisition gradient echo images were acquired with the TR = 2.3 s, TE = 29.8 ms, flip angle = 9°, dimension = 160 × 240 × 256 voxels, and 1 × 1 × 1 mm voxel size.

Diffusion-weighted MRI (DWI) data were acquired with the neurite orientation dispersion and density imaging (NODDI) technique, using the following protocol: TR = 9.4 s, TE = 105 ms, flip angle = 90°, dimension 128 × 128 × 66 voxels, 2 × 2 × 2 mm voxel size, and 2 mm spacing between slices, 100 frames: 10 at b = 0 s/mm^2^, 30 at b = 700 s/mm^2^, 60 at b = 2000s/mm^2^.

Rs-fMRI data were acquired with a T2*-weighted echo planar imaging sequence (TR = 2 s, TE = 30 ms, flip angle = 80°, dimension 64 × 64 × 35 voxels, 3 × 3 × 3 mm voxel size and 3 mm spacing between slices, 300 frames).

Glucose brain metabolism was assessed using the ^18^F-fluorodeoxyglucose positron emission tomography (^18^F-FDG PET/CT) scanning at resting state on a PET/CT scanner (Biograph64 Vision 600, Siemens, Erlangen, Germany). Before radiotracer injection, 20 min of sensorimotor rest were respected in a dark and quiet room. The static ^18^F-FDG PET/CT images were acquired 30 min post-injection (3 MBq/kg ^18^F-FDG) with a 3-dimensional static emission for 16 min. The PET scan attenuation was corrected with the information provided by the CT (120 kVp, 40 mA, FOV 50 cm). Images were reconstructed on a 440 × 440 matrix (PSF + TOF 12i5s), 164 slices, pixel size = 0.825 × 0.825, thickness = 1.6 mm.

### Neuroimaging data preprocessing and derivation of biomarkers’ values

The neuroimaging data were first evaluated for the image quality, and the scans that exceeded the quality control threshold were excluded from the analyses.

The DWI data were denoised, preprocessed, and used to derived fractional anisotropy (FA) maps as described previously in Pozeg et al. [23]. We quantified the structural connectivity of the forebrain mesocircuit using the multi-scale probabilistic atlas of human connectome [56]. We calculated the mesocircuit structural connectome by extracting the mean FA values across the voxels belonging to the white matter bundles connecting the bilateral regions forming a part of the forebrain mesocircuit (the frontal cortex, precuneus, cingulate cortex, thalamic nuclei, and the basal ganglia). We derived the biomarker of structural connectivity (mesocircuit FA) by averaging the FA values across the entire mesocircuit connectome. The method used to derive the mean structural connectivity value is described in detail in the Supplemental Information.

The anatomical and rs-fMRI data were preprocessed using the default fmriprep pipeline (21.0.2) [57, 58]. Resting-state functional connectivity was assessed with the data-driven, group independent component (IC) analysis [59] by decomposing the preprocessed and smoothed data in 20 spatially ICs. We sorted the ICs into the components presenting resting-state networks (RSN) and noise components through the visual inspection of various signal and noise features [60], and through a comparison to the resting-state networks templates [61]. The mean group spatial *t*-value maps of the RSN ICs were thresholded at *t* > 4 and used as brain masks to extract the individual mean spatial map connectivity value (*t*-value) from each patient’s corresponding IC spatial map. Second, we calculated the within-network connectivity of the DMN by correlating the IC time courses of the DMN components. In the same manner, we also calculated the connectivity (anti-correlations) between the DMN and the executive functioning related networks (EFN) [62].

**Fig. 2:**
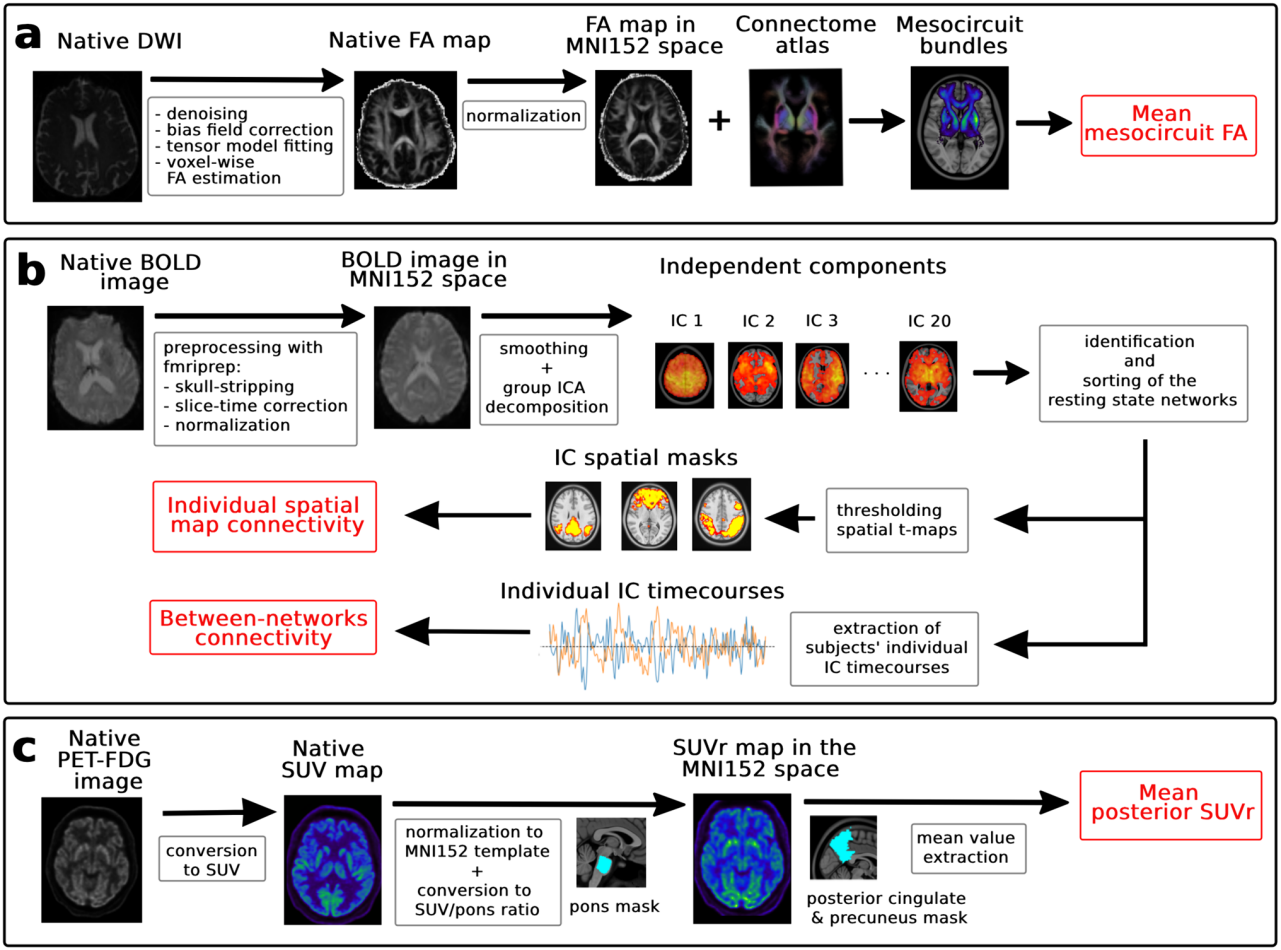
Pre- and post-processing of multimodal neuroimaging data and measures of brain glucose metabolism, structural, and functional connectivity. **a** Native diffusion-weighted image (DWI) was preprocessed and used to derive a fractional anisotropy (FA) map in native space, which was then normalized to the MNI152 space. The atlas tractography was overlaid with the patient’s normalized FA map. The structural connectivity biomarker, the mesocircuit FA, was calculated by averaging the FA values across the voxels belonging to the white matter bundles connecting the brain regions within the anterior forebrain mesocircuit. **b** The native rs-fMRI BOLD images were preprocessed and normalized to the MNI152 space with the fmriprep pipeline. The smoothed images were then analyzed with the group independent component analysis (ICA) and decomposed into 20 independent spatial components. These components were then sorted into the resting-state networks and noise. The average spatial maps of the resting-state networks components were thresholded at *t*-value > 4 and used as masks to extract individual spatial connectivity values. In addition, we extracted the time course signal of the posterior default mode network and dorsal attention network to calculate the between-networks functional connectivity (DMN-DAN anti-correlation). **c** Native static ^18^F-FDG PET images in bq/ml were converted into standard uptake values (SUV) maps. The SUV maps were normalized to the MNI152 space. Each patient’s SUV map was then normalized by the mean value of the pons using the anatomical mask to obtain the SUV ratio (SUVr) map. To obtain the biomarker of the brain metabolism in the posterior cingulate and precuneus (posterior SUVr), we averaged the SUVr value using an anatomical mask for this region of interest

The ^18^F-FDG PET/CT images (in bq/ml) were transformed into the standard uptake values (SUV) maps considering the patient’s body weight and dose decay correction. The SUV maps were co-registered with the native anatomical images and normalized to the MNI space. For each patient, the SUV map was normalized by the mean value of the pons to obtain the SUV ratio (SUVr) map. Mean global SUVr value was calculated across the entire brain grey matter, and the mean posterior SUVr value was derived by averaging the SUVr of the posterior cingulate and the precuneus. The neuroimaging preprocessing and biomarkers’ values extraction steps are detailed in the Supplemental Information and illustrated in Fig. 2.

### Statistical analysis

#### Outcome index

To reduce data dimensionality, we used principal component analysis on patients’ clinical evaluation scores at discharge, combining DRS, RLAS, and FAC scores. The first principal component, reflecting the most explained variance, was defined as the outcome index, representing overall functional and cognitive recovery from coma. We linearly transformed outcome index scores to a positive scale for clarity, where higher scores indicate more favorable outcomes. The analysis details are presented in the Supplemental Information.

#### Univariate regressions and linear regression model comparisons

First, we have tested the strengths of associations between the neuroimaging biomarkers and the outcome index measured at the discharge using Pearson’s correlation coefficient. Then, we compared different nested and non-nested linear regression models to determine which neuroimaging biomarkers can best explain the variance of the outcome index and if they can significantly increase the explained variance of the CRS-R score alone. We first built a minimal “clinical” linear model consisting of the CRS-R score at the approximate time of the MRI and PET scans, and the patient’s age, sex, time between the injury and outcome evaluation at the discharge, and the time between the CRS-R and outcome evaluation at the discharge as confounding variables in the model. This “clinical” linear model was then compared to the non-nested minimal “neuroimaging” linear models, containing each of the neuroimaging biomarkers and the corresponding confounding variables using the Vuong test [63] for non-nested models.

In addition, we evaluated if a simple lesion assessment based on anatomical MRI scan can outperform or improve the minimal clinical linear model. To this end we evaluated the lesion load as described in our previous work [64]. An experienced neuroradiologist assessed bilaterally four cortical (frontal, temporal, parietal, and occipital lobe) and five subcortical regions (basal ganglia, thalamus, mesencephalon, pons, and cerebellum). Each region was scored binary: with 0 when no lesion or a smaller lesion was present and with 1, when a larger focal lesion, covering more than 30% of the region’s volume or a diffuse lesion was present. The lesion load was defined as the sum of the lesion scores for all 18 regions and was included as a predictor in the minimal “lesion” model together with the confounding variables.

We subsequently compared the nested minimal “clinical” linear model to more complex linear models containing additional lesion or neuroimaging biomarkers. The nested linear models were compared and evaluated for the model fit using the Akaike Information Criteria (AIC) as well as the χ^2^ test on log likelihood ratios. The family-wise error rate was controlled by employing Bonferroni method to adjust the *α* level within each family of tests.

## Results

### Subjects

In total, 32 patients (11 women) with age range between 18 and 76 years (*M* = 44.8 years, SD = 17.7) were included in the study. The etiology of brain injury was traumatic (*n* = 15), hemorrhagic (*n* = 6), ischemic (*n* = 1), postanoxic (*n* = 3), SARS-CoV-2-related encephalopathy (*n* = 5), encephalitic (*n* = 1), and other (global rostral midbrain syndrome and corpus callosum infarction in the context of insufficient shunt drainage, *n* = 1).

All patients were identified as cCMD based on the MBT-r tool (i.e., they all showed signs of conscious perception at the first evaluation). The median CRS-R score prior to or at the admission to the unit was 6 (range: 0–23, IQR = 3) and the median score at the approximate time of the scan was 19 (range: 3–23, IQR = 11). The mean outcome index computed based on the RLAS, DRS, and FAC scores at the discharge from the unit was 5.0 (range: 1.3–7.8, SD = 1.6). The summary of the patients’ demographic and clinical data is presented in Table 1. The flow diagram showing patient selection and number of patients per neuroimaging modality is shown in Fig. 1.

**Table 1:** Patients’ demographics and clinical info. ANR: Acute NeuroRehabilitation

Variable			Shapiro–wilk test of normality
Age (years)	18–76, *M* = 44.9 ± 17.8		*W* = 0.95 *p* = 0.16
Sex	Women	11 (34%)	
	Men	21 (66%)	
Etiology	Traumatic	15 (47%)	
	Hemorrhagic	6 (19%)	
	Ischemic	1 (3%)	
	Postanoxic	3 (9%)	
	SARS-CoV-2 encephalopathy	5 (16%)	
	Encephalitic	1 (3%)	
	Other	1 (3%)	
MBT-r classification	cCMD	32 (100%)	
CRS-R diagnosis prior to/at the ANR admission	coma	5 (16%)	
	UWS	15 (47%)	
	MCS	12 (37%)	
CRS-R initial score prior to/at the ANR admission	0–17, *Mdn* = 6, *IQR* = 3		*W* = 0.93 *p* = 0.043
CRS-R score at scan	3–23, *Mdn* = 19, *IQR* = 11		*W* = 0.86 *p* < 0.001
Time between injury and outcome evaluation (days)	23–160, *Mdn* = 57, *IQR* = 27.3		*W* = 0.88 *p* = 0.002
Time between injury and admission to the ANR (days)	3–104, *Mdn* = 23, *IQR* = 17.8		*W* = 0.81 *p* < 0.001
Time between CRS-R initial score and outcome evaluation (days)	13–88, *M* = 43.1, SD = 16.5		*W* = 0.97 *p* = 0.43
Time between CRS-R at scan and outcome evaluation (days)	0–63, *M* = 24.3, SD = 13.5		*W* = 0.96 *p* = 0.20
Time between ^18^F-FDG PET/CT scan and outcome evaluation (days) *n* = 29	2–60, *M* = 23.3, SD = 13.1		*W* = 0.96 *p* = 0.33
Time between DWI scan and outcome evaluation (days) *n* = 24	1–45, *M* = 19.3, SD = 11.7		*W* = 0.95 *p* = 0.29
Time between rs-fMRI scan and outcome evaluation (days) *n* = 23	3–59, *M* = 23.1, SD = 14.9		*W* = 0.94 *p* = 0.17
Outcome index (*n* = 32)	1.3–7.8, *M* = 5, SD = 1.6		*W* = 0.97 *p* = 0.53

*P*-value < 0.05 was considered significant

### Lesion biomarker

The median lesion load score was 3 (range: 0–12, IQR = 4.25). The correlation between the lesion count and the outcome index at the discharge was not significant (Shapiro–Wilk W = 0.87, *p* = 0.001; Spearman’s *ρ* = − 0.25, *p* = 0.168).

### Neuroimaging biomarkers

We did not acquire DWI scans for six subjects due to excessive agitation in the scanner, and the DWI data of two subjects were excluded from the subsequent analysis due to insufficient image quality (movement artifacts). In total, we analyzed the DWI data of 24 patients. The association between the mean mesocircuit FA and the outcome index was strong and significant (Shapiro–Wilk* W* = 0.92, *p* = 0.06; Pearson’s* r* = 0.72, *p* < 0.001, 95% CI: 0.87, 0.45), indicating more preserved white matter fiber integrity of the mesocircuit in patients with better clinical recovery.

We did not acquire rs-fMRI scans for five subjects due to excessive agitation in the scanner, and the rs-fMRI data of subjects was excluded from the subsequent analysis due to insufficient image quality (movement artifacts, *n* = 1), larger brain deformation preventing image co-registration and normalization (*n* = 2), and a different rs-fMRI protocol (*n* = 1). In total, the rs-fMRI data of 23 patients were analyzed. Following the IC decomposition, we identified 13 ICs representing RSN source signals; these were two DMN components: posterior DMN (p-DMN), anterior DMN (a-DMN); four EFNs: dorsal attention (DAN), salience, executive control, and right fronto-parietal network; six primary sensory and motor networks, and the reward network. The mean spatial maps of the DMN and EFN independent components, and their associations with the outcome index are shown in the Supplemental Information, Fig. S3.

The highest correlation between the mean spatial connectivity and the outcome index was found for the p-DMN (Shapiro–Wilk* W* = 0.97, *p* = 0.75; Pearson’s* r* = 0.43, *p* = 0.040, 95% CI: 0.72, 0.02); however, it did not survive the Bonferroni corrected significance level (*α* = 0.0045). The association between the DAN network and the outcome index (Shapiro–Wilk* W* = 0.96, *p* = 0.49, Pearson’s* r* = 0.39, *p* = 0.07, 95% CI: 0.69, − 0.03) was fair/weak and not significant. The other ICs demonstrated weaker and insignificant associations with the outcome index (all *r* < 0.35, *p* > 0.05).

The analyses of within- and between-ICs connectivity demonstrated that the connectivity between the p-DMN and DAN ICs was strongly negatively and significantly associated with the outcome index (Shapiro–Wilk* W* = 0.98, *p* = 0.82; Pearson’s* r* = − 0.74, *p* < 0.001, 95% CI − 0.46, − 0.88). In other words, the patients with stronger negative functional connectivity (anti-correlation) between the p-DMN and DAN showed more favorable clinical indices of recovery. On the other hand, the strength of within-DMN network connectivity (between a-DMN and p-DMN) did not show any association with the outcome index (Shapiro–Wilk* W* = 0.95, *p* = 0.24; Pearson’s* r* = 0.08, *p* = 0.71, 95% CI: 0.48, − 0.34). The connectivity values between the p-DMN and other EFN were weak and did not show any statistically significant correlation with the outcome index (all *p* > 0.05); the scatter plots representing their associations with the outcome index are shown in Supplemental material Fig. S3.

**Fig. 3:**
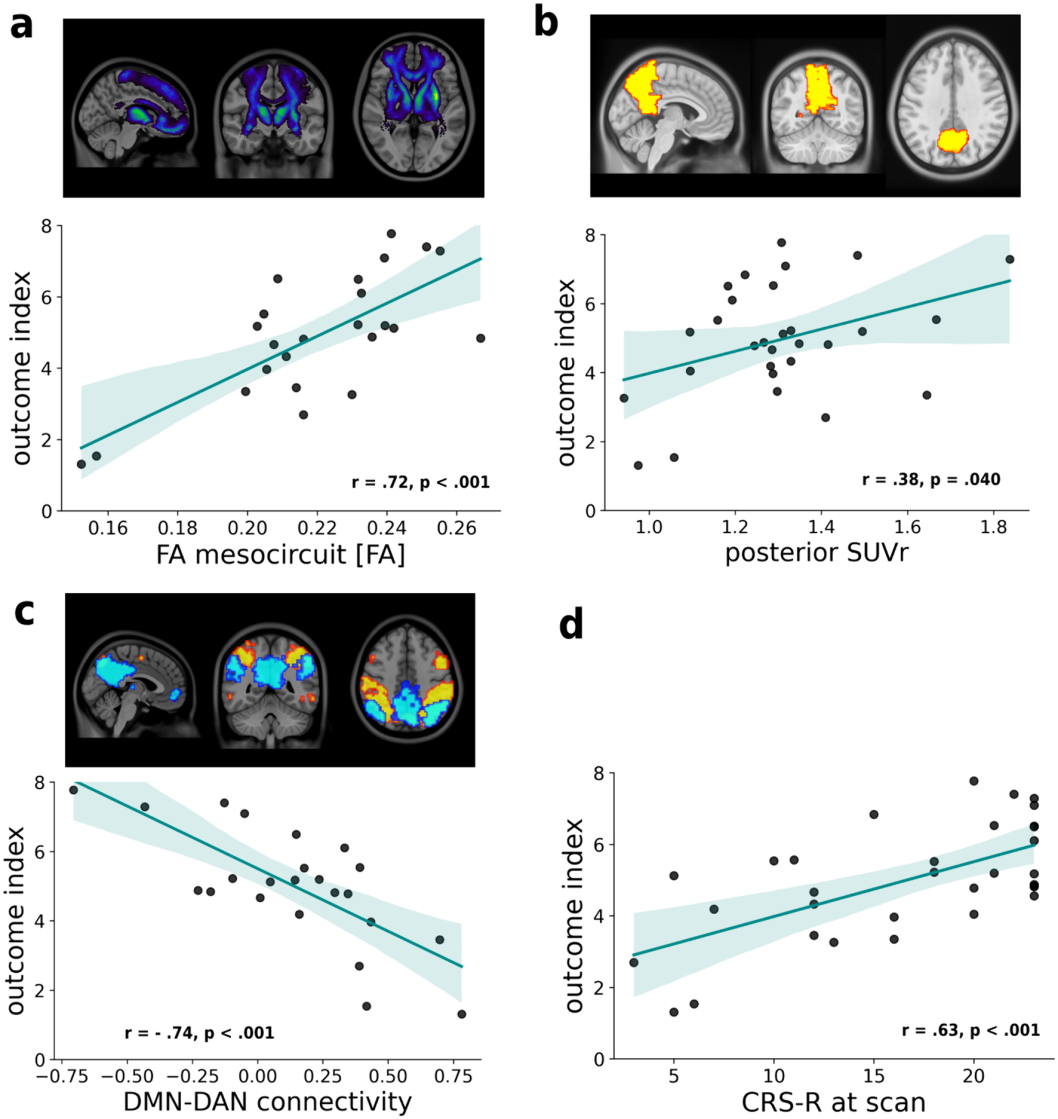
Outcome index and its correlation with neuroimaging biomarkers at the time of discharge. **a** *Upper*: The probabilistic white matter fiber bundles of the anterior forebrain mesocircuit extracted from the human connectome atlas. *Lower*: scatter plot showing the correlation between the mean fractional anisotropy of mesocircuit and the outcome index. **b** *Upper*: the brain mask of the posterior cingulate and precuneus used to extract the mean posterior SUVr value. *Lower:* scatter plot showing the correlation between the posterior SUVr and the outcome index.** c** *Upper*: brain mask representing the resting-state networks of the posterior default mode (DMN) in blue and dorsal attention (DAN) in yellow obtained with the group independent component analysis by thresholding the component’s average spatial *t*-map. *Lower:* the scatter plot is showing the correlation between the outcome index and the negative functional connectivity between the DMN and DAN (anti-correlation). **d** Scatter plot showing the correlation between the Total Coma Recovery Scale—Revised (CRS-R) score and the outcome index. The shaded areas represent the 95% confidence interval of the fitted line

The ^18^F-FDG PET/CT scans could not be acquired for two patients due to their excessive agitation in the scanner. One patient was scanned with a different scanning protocol; therefore, their ^18^F-FDG PET/CT data were not included in the analyses. In total, we analyzed the data of 29 patients. The associations between the mean global SUVr and the outcome index (Shapiro–Wilk* W* = 0.94, *p* = 0.12; Pearson’s* r* = 0.18, *p* = 0.34, 95% CI 0.52, − 0.20) were weak and statistically not significant. The association between the posterior SUVr and the outcome index was fair/weak and significant (Shapiro–Wilk* W* = 0.95, *p* = 0.15; Pearson’s* r* = 0.38, *p* = 0.040, 95% CI: 0.66, 0.02). The scatter plots displaying the most pertinent correlations between neuroimaging biomarkers and the outcome index are shown in Fig. 3.

**Fig. 4:**
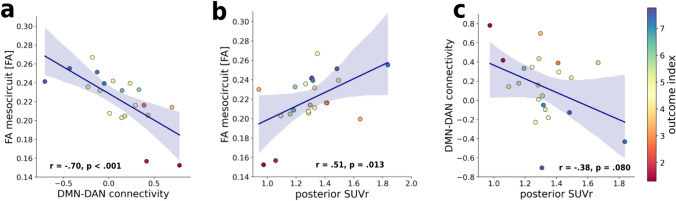
Correlations between neuroimaging biomarkers. **a** Scatter plot representing the correlation between the mean fractional anisotropy value of the mesocircuit and the negative functional connectivity between the default mode and dorsal attention network. **b** Scatter plot representing the correlation between and the negative functional connectivity between the default mode and dorsal attention network and the posterior standard uptake value ratios (SUVr). **c** Scatter plot representing the correlation between the mean fractional anisotropy value of the mesocircuit and the posterior SUVr values. The shaded areas represent the 95% confidence interval of the fitted line. The color bar represents the outcome index value

The mean FA of the mesocircuit significantly correlated with both the negative functional DMN-DAN connectivity (*n* = 20, Pearson’s* r* =  − 0.70, *p* < 0.001, 95% CI: − 0.87, − 0.37) and the posterior SUVr (*n* = 23, Pearson’s* r* = 0.51, *p* = 0.013, 95% CI: 0.13, 0.76), whereas the correlation between the posterior SUVr and the negative functional DMN-DAN connectivity was weaker and statistically not significant (*n* = 22, Pearson’s* r* =  − 0.38, *p* = 0.08, 95% CI: − 0.69, 0.05). The scatter plots displaying correlations between the biomarkers are shown in Fig. 4.

### Linear regression model comparisons

We conducted statistical comparisons on linear models featuring single clinical or neuroimaging predictors, as well as their combinations, while accounting for confounding variables. This analysis was restricted to the subset of patients with neuroimaging data from all three modalities (*n* = 19).

The minimal clinical linear model incorporating only the CRS-R score and the covariates (age, sex, time between the injury and outcome index evaluation, and time between the CRS-R and the outcome index evaluation) explained 75% of variance (*adjusted R*^2^, *p* < 0.001, AIC = 55.0). The CRS-R score significantly predicted the outcome index (*B* = 0.23, *p* < 0.001). However, to note, this association weakened with a longer period between assessments, i.e., between CRS-R at/prior to admission and outcome index at the discharge (*r* = 0.06, *p* = 0.73). Further details and additional analysis including the CRS-R initial score and diagnosis are available in the Supplemental Information.

The minimal lesion model with the lesion load as the single predictor and covariates explained 26% of variance (*adjusted R*^2^) in the outcome index and was statistically not significant (*p* = 0.11, AIC = 75.7).

Statistically significant was the minimal neuroimaging linear model with the functional p-DMN-DAN anti-correlation as the single predictor and the covariates (*adjusted R*^2^ = 0.68, *p* < 0.001, AIC = 59.8), where the p-DMN-DAN anti-correlation significantly predicted the outcome index (*B* =  − 4.0, *p* < 0.001), and the minimal neuroimaging linear model with the structural connectivity biomarker (*adjusted R*^2^ = 0.65, *p* = 0.002, AIC = 61.6), where the mesocircuit FA value significantly predicted the outcome index at the discharge (*B* = 46.1, *p* = 0.001).

Comparing the linear models’ explained variance and AIC, none of the minimal lesion or neuroimaging linear models outperformed the clinical linear model. The Vuong test for comparison of non-nested models confirmed the clinical model’s superior goodness of fit (all *p* > 0.05).

**Fig. 5:**
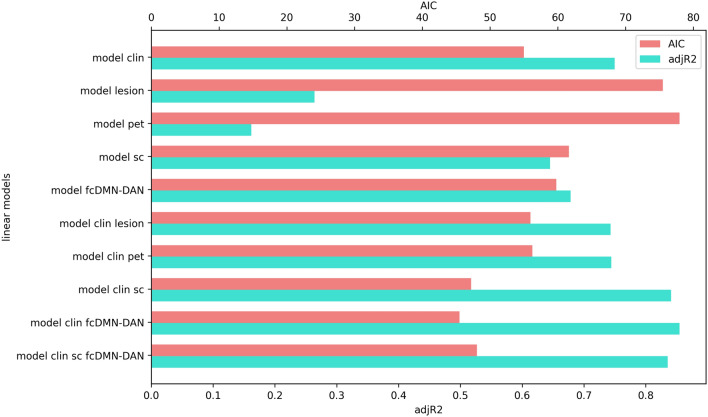
Linear models’ performance comparison. Bar chart showing the models performance metrics: explained variance with adjusted *R*^2^ (blue) and Akaike information criterion (AIC, red). Minima linear models: clin = minimal clinical, lesion = minimal lesion pet = minimal ^18^F-FDG PET/CT, sc = minimal structural, fcDMN-DAN = minimal functional; Nested linear models: clin lesion = clinical + lesion, clin pet = clinical + ^18^F-FDG PET/CT, clin sc = clinical + structural, clin fcDMN-DAN = clinical + functional, clin sc fcDMN-DAN = clinical + structural + functional

Statistical comparison of the nested models showed that adding the structural connectivity biomarker (mesocircuit FA) to the clinical linear model significantly improved the model fit (*adjusted R*^2^ = 0.84, *χ*^2^ = 11.8, *p* = 0.003). The prediction of the outcome was also significantly improved when adding the anti-correlation between the p-DMN and DAN as the fMRI biomarker to the clinical linear model (*adjusted R*^2^ = 0.85, *χ*^2^ = 13.5, *p* = 0.001). However, addition of both significant biomarkers at once (the mesocircuit FA and the functional DMN-DAN anti-correlation) did not further improve the prediction of the outcome (*adjusted R*^2^ = 0.84, *χ*^2^ = 3.1, *p* > 0.05). Other neuroimaging biomarkers did not show statistically significant improvement of the goodness of fit (all *p* > 0.05). The statistical tests of model comparisons are shown in Table 2. The linear models’ performance is graphically displayed in Fig. 5.

**Table 2:** Model comparisons statistics

Prediction of the outcome index at discharge from the acute neurorehabilitation unit						
Model	Adj. R^2^	AIC	Model F (*p* value)	Predictor *t* (*p* value)	Vuong test *z* (*p* value)	χ^2^ test (*p* value)
Minimal clinical						
**CRS-*R*** + age + sex + time: injury to outcome evaluation + time: CRS-R to outcome evaluation	0.75	55.0	11.8 (< 0.001)	6.5 (< 0.001)		–
Minimal lesion						
**Lesion load** + age + sex + time: injury to outcome evaluation + time: CRS-R to outcome evaluation	0.26	75.7	2.3 (0.11)	− 2.4 (0.035)	2.62 (> 0.99)	–
Minimal structural						
**FA mesocircuit** + age + sex + time: injury to outcome evaluation + time: dMRI to outcome evaluation	0.65	61.6	7.5 (0.002)	4.6 (0.001)	0.91 (0.82)	–
Minimal functional						
***p*-DMN-DAN anti-correlation** + age + sex + time: injury to outcome evaluation + time: rs-fMRI to outcome evaluation	0.68	59.8	8.6 (< 0.001)	− 5.5 (< 0.001)	0.68 (0.75)	–
Minimal ^18^F-FDG PET/CT						
**pSUVr** + age + sex + time: injury to outcome evaluation + time: PET to outcome evaluation	0.16	78.0	1.7 (0.21)	1.7 (0.11)	3.5 (> 0.99)	–
Clinical + lesion						
**CRS-*R*** + **lesion load** + age + sex + time: injury to outcome evaluation + time: CRS-R to outcome evaluation	0.74	56.0	9.6 (< 0.001)	5.0 (< 0.001) 0.82 (0.43)	–	1.04 (0.31)
Clinical + structural						
**CRS-*R*** + **FA mesocircuit** + age + sex + time: injury to outcome evaluation + time: CRS-R to outcome evaluation + time: dMRI to outcome evaluation	0.84	47.2	14.6 (< 0.001)	4.2 (0.002) 2.2 (0.048)	–	11.8 (0.003)
Clinical + functional						
**CRS-*R*** + *p***-DMN-DAN anti-correlation** + age + sex + time: injury to outcome evaluation + time: CRS-R to outcome evaluation + time: rs-fMRI to outcome evaluation	0.85	45.5	16.1 (< 0.001)	4.1 (0.002) − 3.0 (0.013)	–	13.5 (0.001)
Clinical + ^18^F-FDG PET/CT						
**CRS-*R*** + **pSUVr** + age + sex + time: injury to outcome evaluation + time: CRS-R to outcome evaluation + time: PET to outcome evaluation	0.74	56.2	8.5 (0.001)	5.5 (< 0.001) 1.0 (0.34)	–	2.8 (0.25)
Clinical + structural + functional						
**CRS-*R*** + **FA mesocircuit** + ***p*-DMN-DAN anti-correlation** + age + sex + time: injury to outcome evaluation + time: CRS-R to outcome evaluation + time: dMRI to outcome evaluation + time: rs-fMRI to outcome evaluation	0.84	48.1	11.2 (< 0.001)	3.3 (0.009) 0.8 (0.45) − 1.3 (0.24)	–	3.1 (0.21)

*P*-value < 0.05 was considered significant

## Discussion

In this prospective study, we investigated diverse multimodal neuroimaging biomarkers to predict recovery after coma. Our focus included the indices of DWI-derived structural connectivity, rs-fMRI-derived functional connectivity, and brain glucose metabolism estimated with the ^18^F-FDG PET/CT imaging. Univariate analysis showed strong and significant associations between the recovery levels and white matter integrity in the anterior forebrain mesocircuit, as well as with functional segregation between the DMN and DAN during rs-fMRI. In multivariate linear regression models, both structural and functional connectivity biomarkers significantly improved the recovery prediction in the post-acute phase.

Our findings demonstrate that patients with stronger structural connectivity in the anterior forebrain mesocircuit display more favorable neurological evolution at the discharge from the acute neurorehabilitation unit. This aligns with the mesocircuit hypothesis, and points to the common underlying neural architecture, necessary for the recovery of consciousness [24, 25]. This neural circuit encompasses the frontal cortices and the striato-pallidal negative loop, which regulates the excitatory thalamo-cortical projections [24]. Lesions impacting the circuit cause disfacilitation of the main anterior frontal cortical targets (anterior cingulate and medial frontal cortex) and result in the down regulation of arousal [65, 66]. The hypothesis is supported by similar studies using DWI showing that greater lesion burden of the structures or of the connecting white matter tracts within the mesocircuit is associated with a worse outcome [23, 27, 28, 64, 67–70].

We also showed that the negative functional connectivity (anti-correlation) between the two antagonistic fronto-parietal networks correlates with the degree of recovery from coma. This finding implicates that the patients with better functional and cognitive recovery profiles displayed more preserved intrinsic cortico-cortical organization of the brain, a necessary property for adequate information integration and processing. In line with the previous research [39, 41, 71, 72], our results show that the strength of anti-correlation between the DMN and DAN could be a promising biomarker for the preserved neural capacity to sustain awareness.

Contrary to prior research, our study only partially replicated the common finding of restored within-connectivity of the DMN [20]. We observed a moderate association between the p-DMN spatial map connectivity and the outcome index, but no correlation in the connectivity strength between the posterior and anterior DMN nodes with the degree of recovery. The lack of correlation might be attributed to the medial prefrontal cortex (mPFC), which serves as both the anterior hub of the DMN, and the salience network, and is considered a functionally heterogeneous region, involved in various cognitive and affective processes [73]. Consequently, our analysis might have not reflected the within-DMN connectivity. As preserved within-DMN connectivity was also reported in unresponsive patients and propofol-induced unconscious subjects [39, 74], it is suggested that this connectivity does not exclusively represent conscious mental activity, but rather a fundamental functional brain organization that is necessary, yet not sufficient for sustenance of consciousness [75, 76]. In addition, the outcome in our study was defined with an interval scale based on the multidimensional neurological evaluation, and not on binary classification based on the Glasgow Outcome Scale [77] or CRS-R recovery of consciousness.

While we found a fair/weak association between the glucose metabolism in the posterior cingulate/precuneus and the outcome index, this biomarker has not shown to significantly improve the prediction of the recovery. Although ^18^F-FDG PET/CT imaging previously showed a promising role in diagnosing patients with disorders of consciousness [12, 30, 43, 78], the measure is biased by various factors, such as the use of substances, and artifacts including hyperglycemia, resulting in larger variations in glucose metabolism among the subjects [79]. In addition, as already suggested and also observed in our data, ^18^F-FDG PET/CT imaging might have a higher accuracy to identify patients who will not display any improvement in recovery of consciousness, but a lesser sensitivity to predict recovery in a graded manner [12].

We found significant correlations between the mesocircuit structural connectivity and the DMN-DAN negative functional connectivity, and between the mesocircuit structural connectivity and glucose metabolism in the posterior cingulate/precuneus. This finding further corroborates observations of the interactions between the two system components [25] that are necessary to enable a sufficient arousal of the system through the brainstem-thalamo-cortical projections, and which facilitate adequate communication between high level cortical networks, required for conscious mental activity [41, 68, 80, 81]. Our findings also highlight the important involvement of the posterior cingulate/precuneus in the recovery of consciousness, aligning with previous studies showing reduced functional [82], effective connectivity [83], and metabolic activity [43, 44] within this region in the patients with disorders of consciousness. This brain region displays dense long-range connections with the frontal regions, temporal lobes, parahippocampal areas, and with the pontine regions [84]. Studies demonstrated that the dorsal posterior cingulate/precuneus forms a functional part of the DMN, while its ventral part activates with the central executive network during cognitively demanding tasks [84], suggesting an important modulating role in the interaction between attention and cognition. With its strategic position, it is viewed as a main hub area to integrate internal and external stimuli, and associate them with existing knowledge in order to facilitate an adequate behavioral response [85].

Lastly, we assessed whether incorporating neuroimaging biomarkers improves outcome prediction compared to the CRS-R evaluation alone. None of the neuroimaging biomarkers alone outperforms the CRS-R evaluation. However, adding either the mesocircuit structural connectivity index or the DMN-DAN negative functional connectivity index significantly increased the explained variance in recovery. While the CRS-R remains important for standardized bedside evaluation, there is an overlap with neurobehavioral scales (most notably the DRS) in the measured construct, showing high concurrent validity [3, 86], and therefore, presenting a confounding factor of multicollinearity in the interpretation of the true predictive validity of the scale.

Our study also showed that deploying both structural and functional connectivity biomarkers together does not add additional value to the prediction of the outcome. This has important implications for diagnostic tests planning, especially in settings with limited access to the neuroimaging facilities. When deciding between using the DWI-FA derived structural or the rs-fMRI connectivity, the latter can be affected by the arousal levels and sedation [87], therefore, its validity might be hindered when used in the critical care in the early phase of the injury. In addition, structural connectivity is less prone to time-related changes, and is, unlike functional connectivity, state-independent. As such, it remains a good candidate for the neuroimaging biomarker of recovery from coma. However, a future longitudinal study is essential to confirm its stability and predictive validity across different injury phases and recovery time intervals.

The study has certain limitations, including a small sample size, requiring validation on a larger sample for generalizability. Further research should also explore whether different neuroimaging acquisition protocols, preprocessing pipelines, and noise removal methods yield similar results, ensuring the robustness of findings. Here we have used the FA as a metric for white matter integrity as it is a most commonly used marker of cerebral white matter microstructure. However, its interpretability is reduced in the presence of crossing fibers or edema [88], thus a confirmatory study using advanced diffusion imaging techniques that account for different neurite orientations [89] is needed. In the same line, there is no single way to derive the RSN from the rs-fMRI. We here followed the state-of-the art, open access, and robust pipeline for neuroimaging preprocessing [57] and a commonly used tool for the group independent component analyses [59], in order to facilitate the reproducibility of the current study. Nevertheless, the group ICA RSN spatial maps are inherently sample-dependent. For these reasons, we showed that atlas-based extraction of DMN-DAN inter-network connectivity produces similar results (see Supplemental Information), suggesting a less expertise-demanding and more generalizable approach may be used instead. We acknowledge that reproducibility of neuroimaging studies represents a significant challenge for implementing rs-fMRI-based biomarkers in clinical settings [90]. Standardizing methodology and outcome definitions is crucial to develop reliable and accurate neuroimaging biomarkers for diagnosing and prognosing disorders of consciousness.

In conclusion, our study demonstrates that greater preserved structural connectivity in the anterior forebrain mesocircuit and stronger negative functional rs-fMRI connectivity between DMN and DAN are significantly correlated with a more favorable neurological evolution upon hospital discharge. In multivariate linear regression models, we showed that the individual structural or functional connectivity biomarker, but not their combination, significantly improves the model fit to predict the recovery in the post-acute phase compared solely to the bedside neurobehavioral evaluation. These findings have implications for selecting diagnostic tests, improving the patient identification for potential recovery, planning a targeted therapy, and aiding in life-death decision-making.

## Supplementary information

Supplementary information is available at *Journal of Neurology* online.

## Supplementary Information

Below is the link to the electronic supplementary material.

Supplementary file 1 (DOCX 19479 kb)

## Data Availability

The structural connectomes, spatial masks and time series from the rs-fMRI group independent component analysis and extracted global and posterior SUVr values are available at the open-source public data repository (https://zenodo.org), 10.5281/zenodo.10581959.
