# Supplementary material for: Explaining recovery from coma with multimodal neuroimaging
Source: J Neurol. 2024 Aug 1;271(9):6274–88. doi: 10.1007/s00415-024-12591-y (PMC11377522; doi:10.1007/s00415-024-12591-y)
Supplement: Supplementary file 1 — (DOCX 19479 kb) [file 415_2024_12591_MOESM1_ESM.docx]

Journal of Neurology

Explaining recovery from coma with multimodal neuroimaging

Polona Pozeg,^1^ Jane Jöhr,^2^ John O. Prior,^3^ Karin Diserens,^2,†^ and Vincent Dunet^1,†^

**^†^These authors contributed equally to this work.**

**Author affiliations:**

1 Department of Radiology, Lausanne University Hospital, 1011 Lausanne, Switzerland

2 Acute Neurorehabilitation Unit, Department of Clinical Neurosciences, Lausanne University Hospital, 1011 Lausanne, Switzerland

3 Department of Nuclear Medicine and Molecular Imaging, Lausanne University Hospital, 1011 Lausanne, Switzerland

Correspondence to: Vincent Dunet

Rue du Bugnon 46, 1011 Lausanne, Switzerland

[vincent.dunet@chuv.ch](mailto:vincent.dunet@chuv.ch)

**Supplemental Information**

**1 Neuroimaging data preprocessing and derivation of biomarkers’ values**

**1.1 Diffusion MRI preprocessing and measures of structural connectivity**

The diffusion weighted imaging (DWI) data image quality was assessed visually by an experienced radiologist and with automatic quality control using EDDY QC [1]. Images that exceeded the suggested threshold [2] of two or more quantitative quality metrics (average absolute motion ≥ 2mm, average relative motion ≥ 0.5 mm, total outliers percentage ≥ 2%) were excluded from the analysis.

We used the Connectome Mapper[3] toolbox (v3.0.4) pipeline to preprocess the DWI data: the images in the native space were denoised with the MRtrix workflow[4], bias field-corrected with the Advanced Normalization Tools (ANTs) algorithm [5], and eddy current and motion-corrected with the FSL eddy correct [6]. Fractional anisotropy (FA) maps were derived from the preprocessed DWI images by first fitting a second order tensor model using the MRtrix’s dwi2tensor command and then estimating the voxel-wise FA map using the tensor2metric command. The native anatomical images were non-linearly transformed into the Montreal Neurological Institute (MNI) standard brain template (ICBM 2009c Nonlinear Asymmetric 1x1x1 mm standard space) by using the ANTs’ registration method (antsRegistrationSyN.sh) and the derived transformation matrices were then applied to the native FA maps.

The structural connectivity of the anterior forebrain mesocircuit was calculated by using the multi-scale probabilistic atlas of human connectome [7]. This atlas was constructed from the diffusion MRI of 66 healthy adults whose data is freely available as a part of the Human Connectome Project. The atlas models white matter connectivity of cortical and sub-cortical grey matter regions, parcellated at four different granularity scales based on the Lausanne 2018 parcellation [8]. The atlas probabilistic tractography was overlaid with each individual fractional anisotropy (FA) map and the mean FA values were extracted for each bundle connecting each pair of pre-specified regions within the mesocircuit. In order to threshold the voxels with low probability from a bundle, the calculations were limited only to the connections present in 80% of the population and to the voxels belonging to the bundle in 90% of the subjects. We pre-selected the regions from the scale 1 Lausanne 2018 parcellation atlas. Our selection was guided by the mesocircuit hypothesis of Schiff et al. [9, 10], which proposes the involvement of frontal/prefrontal cortical-striatopallidal thalamocortical loop systems in the regulation of a widespread cortical activity. Our region selection therefore included the following bilateral regions: frontal pole, medial orbito frontal, rostral middle frontal, superior frontal, caudal middle frontal, precentral gyrus, rostral anterior cingulate, pulvinar, ventral anterior thalamus, mediodorsal thalamus, lateral-posterior ventral posterior thalamus, medial pulvinar centrolateral, ventrolateral thalamus, ventral posterior ventrolateral thalamus, caudate, putamen, and globus pallidus. The mean FA of all the bundles connecting these regions was then used as the index of the mesocircuit structural connectivity. The DWI data pre- and post-processing steps are illustrated in Figure 2.

**1.2 Resting-state fMRI (rs-fMRI) preprocessing and measures of functional connectivity**

The quality of the rs-fMRI scans was first evaluated visually and then quantitatively by using the MRI quality control (MRIQC) tool [11]. The scans that exceeded the mean motion of 2mm or where the percentage of the frame-wise displacement was above 50% were excluded from the analysis. The anatomical and rs-fMRI data were preprocessed by using the default fmriprep pipeline (21.0.2) [12, 13], where the rs-fMRI scans were skull-stripped, slice-time corrected, and normalized to the fmriprep’s MNI152 template space. The preprocessed images were then spatially smoothed with a 6-mm full width at half-maximum Gaussian kernel using SPM12 (https://www.fil.ion.ucl.ac.uk/spm/software/spm12/).

Resting-state functional connectivity was assessed with the data-driven, group independent component (IC) analysis (gICA) on the preprocessed and smoothed rs-fMRI data using the Group ICA Of fMRI Toolbox (GIFT, v4.0b; <https://trendscenter.org/software/gift>) [14]. The rs-fMRI data were decomposed into 20 spatially ICs using the infomax algorithm implemented in the GIFT’s toolbox. The corresponding patient-specific spatial maps were then calculated through a back reconstruction step. The ICs were sorted into the default mode (DMN), executive function (task positive) related networks (EFN) [15], sensory networks (SN) and the noise through the visual inspection of various signal and noise features [16], and through a comparison to the resting state networks templates [17]. The ICs identified as noise were removed from the analyses, while the mean group spatial t-value maps of the ICs identified as presenting resting state network (RSN) signals were thresholded at t > 4. These thresholded maps were used as brain masks to extract the individual mean spatial map connectivity value (t-value) from each patient’s corresponding IC spatial map.

The values of the first five frames of each patient RSN IC time courses were removed due to the initial signal intensity instability. The RSN IC time courses were then despiked, detrended, and denoised with 24 head-motion parameters, CSF and white matter signal derived regressors, obtained from the fmriprep confound estimation, and normalized to z-scores, using the “signal.clean” function of nilearn Python package [18]. We calculated the within-network connectivity of the DMN by correlating the IC time courses of the DMN components. In the same manner, we also calculated the connectivity (anti-correlations) between the DMN and the most prominent EFN. The rs-fMRI data pre- and post-processing steps are illustrated in Figure 2.

**1.3 18F-FDG PET/CT image preprocessing and data analysis**

The ^18^F-FDG PET/CT images (in bq/ml) were transformed into the standard uptake values (SUV) maps taking into account the patient’s body weight and dose decay correction using the following formula[19]:

$$SUV =\frac{A_{c}}{D \times e^{-\ln\left( 2 \right) \times\left( \frac{\Delta t}{T_{\frac{1}{2}}} \right)}} \times W$$

where A_c_ = activity concentration (Bq/ml), D = injected dose activity (Bq), Δt = time difference between the injection time and scan time (s), $T_{\frac{1}{2}}$ = half-life of the radionuclide (s), and W = body weight (g).

The SUV maps were co-registered with the native anatomical T1-weighted images and normalized to the MNI space template using the SPM12 toolbox (https://www.fil.ion.ucl.ac.uk/spm/). For each patient, the SUV map was normalized by the mean value of the pons, which was extracted by using a mask derived from the Lausanne2018 atlas parcellation[8] to obtain the SUV ratio (SUVr) map. Mean global SUVr value was calculated across the grey matter using the MNI space grey matter probability mask. Mean SUVr value of the posterior cingulate and the precuneus (posterior SUVr) was extracted from the SUVr map using the mask derived from the AAL atlas parcellation [8]. The ^18^F-FDG PET/CT data pre- and post-processing steps are illustrated in Figure 2.

### 2 Outcome index

To reduce the data dimensionality, and avoid issues with multicollinearity and multiple testing, we created a composite score using principal component analysis on the patients’ clinical neurobehavioral test scores obtained at the discharge from the unit. Patients’ scores from the DRS, RLAS, and FAC were normalized to z-values and then the first principal component accounting for the largest proportion of explained variance was used as the primary outcome – the outcome index, indicating the overall level of functional and cognitive recovery from coma. For a clearer representation, the outcome index scores were linearly transformed to a positive scale, where a higher outcome index indicated a more favorable outcome. The first component obtained using the principle component analysis explained 81% of variance in the data. The loadings of the DRS, RLAS, and FAC on the first component are shown in Fig.S1.

**3 CRS-R correlation with the outcome**

The initial CRS-R total score taken prior to/at the admission to the acute neurorehabilitation unit did not significantly correlate with the outcome index at the discharge from the unit (*n* = 32, *Pearson’s r* = 0.06, *p* = 0.73, *95% CI*: -0.29, 0.40). The CRS-R total score taken at the time of a scan significantly correlated with the outcome index at the time of discharge (*n* = 32, *Pearson’s r* = 0.63, *p* < .001, *95% CI*: 0.36, 0.80). The outcome index at the discharge did not significantly differ between the Coma/UWS (*M* = 5.06, *SD* = 1.66) and MCS (*M* = 4.91, *SD* = 1.52) patients diagnosed based on the CRS-R criteria prior to the admission to the neurorehabilitation unit (*Student’s t*(30) = 0.25, *p* = 0.80, *d* = 0.09). The distribution of the outcome index at discharge for Coma/UWS and MCS patients groups based on the initial CRS-R diagnosis are shown in Fig.S2.

We compared the “early” clinical models including the initial CRS-R total score or the classification based on the CRS-R diagnosis evaluated prior to the admission to the acute neurorehabilitation unit with the clinical model when the CRS-R total score was performed at the time of a scan. All the models included age, sex, and the time between the CRS-R and the outcome index evaluation as covariates. The early clinical model including the initial CRS-R total score explained 15% of the outcome index variance and was not statistically significant (*adjusted R^2^*, *AIC* = 78.3, *p* =0.22). The initial CRS-R total score in the model did not significantly predict the outcome index (*B* = 0.29, *p* = 0.09). Similarly, the early clinical model including the CRS-R diagnosis classification was statistically insignificant (*adjusted R^2^* = -0.04, *AIC* = 82.2, *p* = 0.54), where the CRS-R classification as a predictor was not statistically significant (*B* = 0.64, *p* = 0.60). Neither of the two “early” models outperformed the clinical model with the CRS-R taken at the time of the scan (Vuong test for non-nested models: *z* = 2.56, *p* = 0.99, and *z* = 2.59, *p* > 0.99, respectively).

**4 Atlas-based extraction of the DMN-DAN inter-network connectivity**

The preprocessed rs-fMRI images in the MNI space (see Methods) were analyzed using the Python’s Nilearn NiftiMasker package [18]. The rs-fMRI images were smoothed with a 6-mm full width at half-maximum Gaussian kernel. We used the Yeo’s 2011 atlas of 7 resting state networks [17] to extract the mean time courses for the DMN and the dorsal attention network (DAN) for each subject. We applied a temporal high-pass filter with a cut-off at 0.0001 and low-pass filter with a cut-off at 0.15 Hz. The first five frames of each patient’s time course values were removed due to the initial signal intensity instability and were then denoised with 24 head-motion parameters, cerebro-spinal fluid and white matter signal derived regressors and normalized to z-scores. The correlation between the atlas-based DMN and DAN time courses were then calculated for each patient and used as the index for the functional DMN-DAN connectivity.

The atlas-based DMN-DAN connectivity significantly correlated with the outcome index at the discharge (*n = 23*, *Shapiro Wilk W* = 0.97, *p* = 0.72, *Pearson’s r* = -0.54, *p* = 0.008, *95% CI*: -0.78, -0.16). The scatterplot depicting the association between the atlas-based DMN-DAN functional connectivity and the outcome index is shown in Figure S3.

**References**

1. Bastiani M, Cottaar M, Fitzgibbon SP, et al (2019) Automated quality control for within and between studies diffusion MRI data using a non-parametric framework for movement and distortion correction. NeuroImage 184:801–812. https://doi.org/10.1016/j.neuroimage.2018.09.073

2. RPubs - Automated QC of SPINS DWI with `eddy quad` and `MRTrix`. https://rpubs.com/navona/SPINS_DWI_QCautomated. Accessed 20 Jan 2023

3. Tourbier S, Rue-Queralt J, Glomb K, et al (2022) Connectome Mapper 3: A Flexible and Open-SourcePipeline Software for Multiscale Multimodal Human ConnectomeMapping. J Open Source Softw 7:4248. https://doi.org/10.21105/joss.04248

4. Tournier J-D, Smith R, Raffelt D, et al (2019) MRtrix3: A fast, flexible and open software framework for medical image processing and visualisation. NeuroImage 202:116137. https://doi.org/10.1016/j.neuroimage.2019.116137

5. Tustison NJ, Avants BB, Cook PA, et al (2010) N4ITK: Improved N3 Bias Correction. IEEE Trans Med Imaging 29:1310–1320. https://doi.org/10.1109/TMI.2010.2046908

6. Jenkinson M, Beckmann CF, Behrens TEJ, et al (2012) FSL. NeuroImage 62:782–790. https://doi.org/10.1016/j.neuroimage.2011.09.015

7. Alemán-Gómez Y, Griffa A, Houde J-C, et al (2022) A multi-scale probabilistic atlas of the human connectome. Sci Data 9:516. https://doi.org/10.1038/s41597-022-01624-8

8. Cammoun L, Gigandet X, Meskaldji D, et al (2012) Mapping the human connectome at multiple scales with diffusion spectrum MRI. J Neurosci Methods 203:386–397. https://doi.org/10.1016/j.jneumeth.2011.09.031

9. Schiff ND (2010) Recovery of consciousness after brain injury: a mesocircuit hypothesis. Trends Neurosci 33:1–9. https://doi.org/10.1016/j.tins.2009.11.002

10. Schiff ND (2023) Mesocircuit mechanisms in the diagnosis and treatment of disorders of consciousness. Presse Médicale 52:104161. https://doi.org/10.1016/j.lpm.2022.104161

11. Esteban O, Birman D, Schaer M, et al (2017) MRIQC: Advancing the automatic prediction of image quality in MRI from unseen sites. PLOS ONE 12:e0184661. https://doi.org/10.1371/journal.pone.0184661

12. Esteban O, Markiewicz CJ, Blair RW, et al (2019) fMRIPrep: a robust preprocessing pipeline for functional MRI. Nat Methods 16:111–116. https://doi.org/10.1038/s41592-018-0235-4

13. Esteban O, Ciric R, Finc K, et al (2020) Analysis of task-based functional MRI data preprocessed with fMRIPrep. Nat Protoc 15:2186–2202. https://doi.org/10.1038/s41596-020-0327-3

14. Calhoun VD, Adali T, Pearlson GD, Pekar JJ (2001) A method for making group inferences from functional MRI data using independent component analysis. Hum Brain Mapp 14:140–151. https://doi.org/10.1002/hbm.1048

15. Witt ST, van Ettinger-Veenstra H, Salo T, et al (2021) What Executive Function Network is that? An Image-Based Meta-Analysis of Network Labels. Brain Topogr 34:598–607. https://doi.org/10.1007/s10548-021-00847-z

16. Griffanti L, Douaud G, Bijsterbosch J, et al (2017) Hand classification of fMRI ICA noise components. NeuroImage 154:188–205. https://doi.org/10.1016/j.neuroimage.2016.12.036

17. Thomas Yeo BT, Krienen FM, Sepulcre J, et al (2011) The organization of the human cerebral cortex estimated by intrinsic functional connectivity. J Neurophysiol 106:1125–1165. https://doi.org/10.1152/jn.00338.2011

18. Abraham A, Pedregosa F, Eickenberg M, et al (2014) Machine learning for neuroimaging with scikit-learn. Front Neuroinformatics 8:

19. Shin H-B, Sheen H, Lee H-Y, et al (2017) Digital Imaging and Communications in Medicine (DICOM) information conversion procedure for SUV calculation of PET scanners with different DICOM header information. Phys Med 44:243–248. https://doi.org/10.1016/j.ejmp.2017.05.063


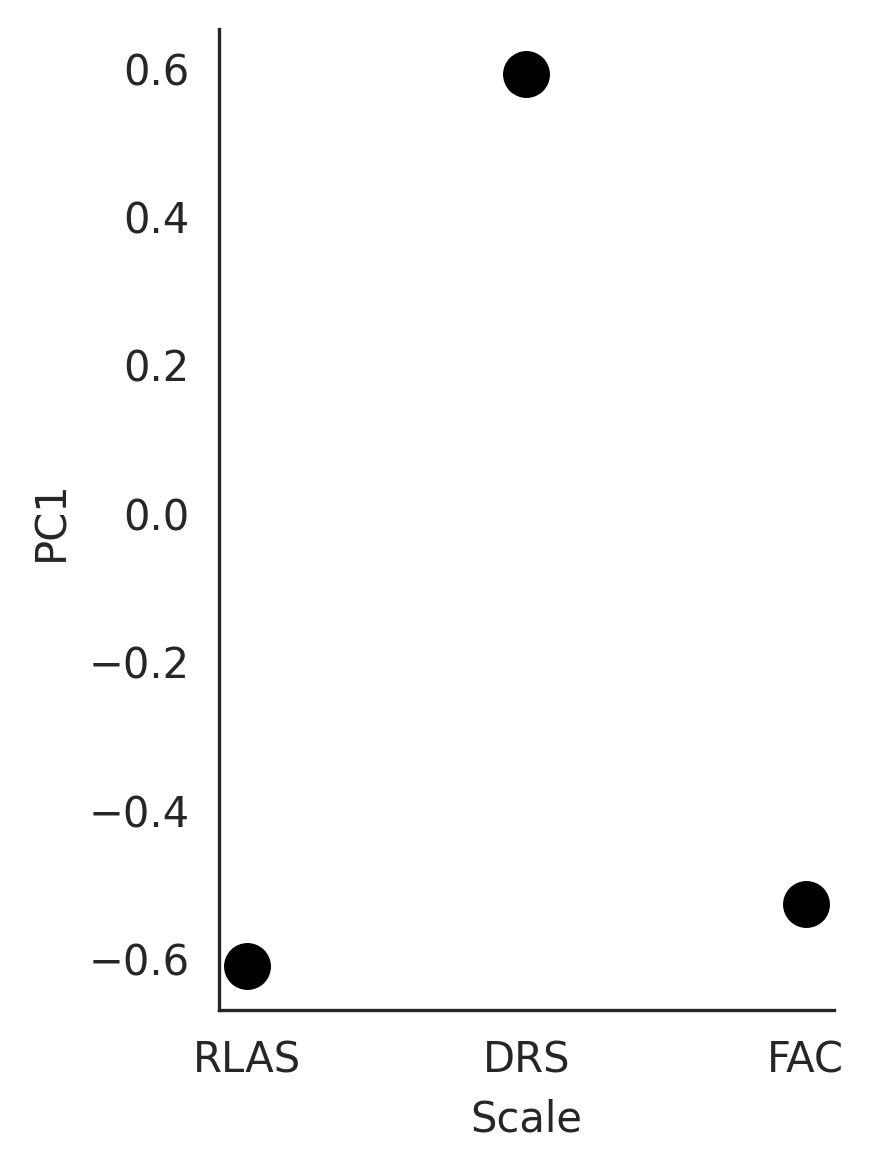


**Fig.S1 Loadings of the outcome clinical variables on the first principal component.** Plot showing the loadings of the Ranchos Los Amigos Level of Cognitive Functioning Scale (RLAS), Disability Rating Scale (DRS), and the Functional Ambulation Categories (FAC), measured at the discharge from the acute neurorehabilitation unit.


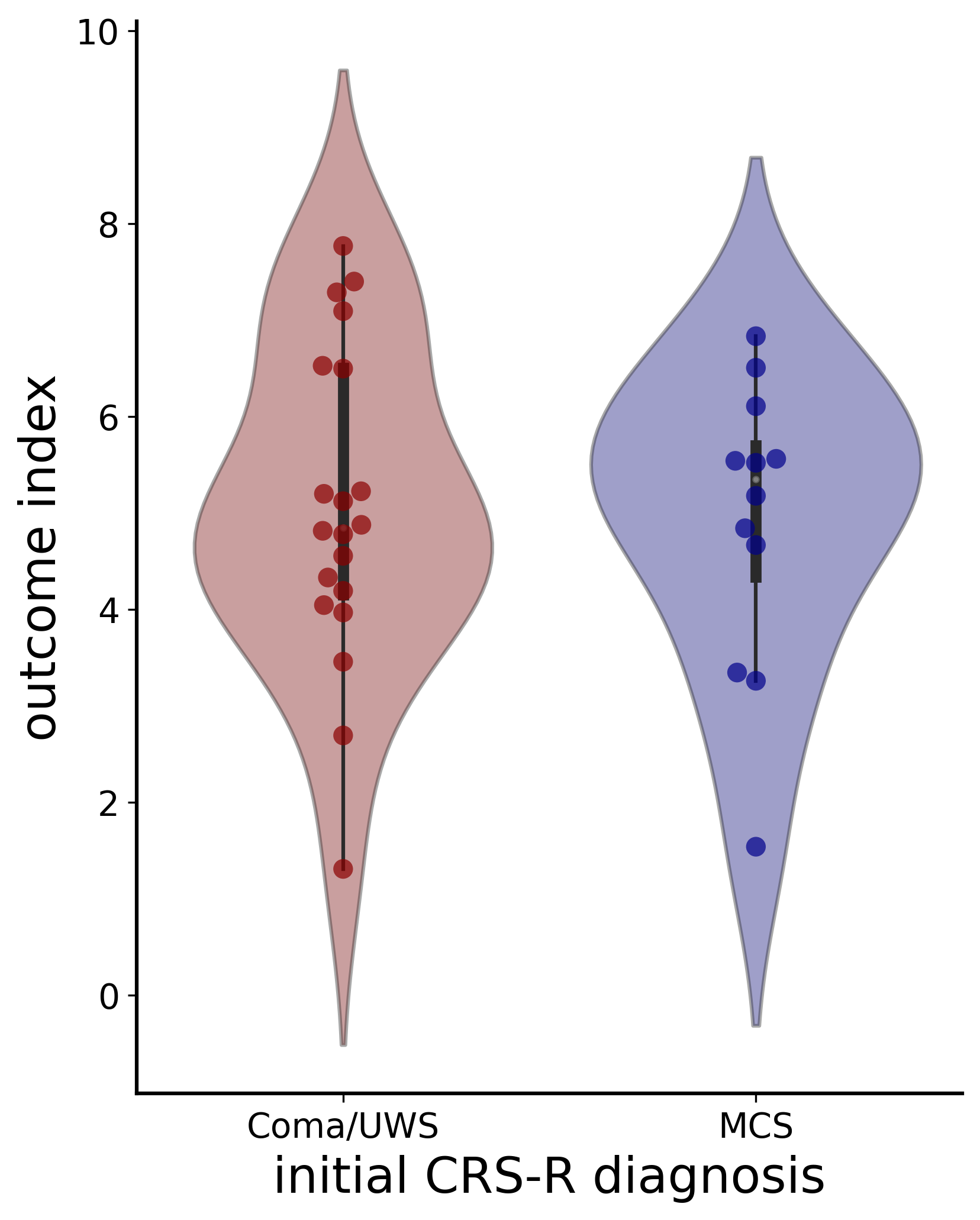


**Fig.S2. Outcome index of the Coma/UWS and MCS patients.** Violin plots showing the outcome index evaluated at the discharge from the acute neurorehabilitation unit for the patients who were diagnosed with Coma/UWS or MCS based on the CRS-R prior to/at the admission to the unit.


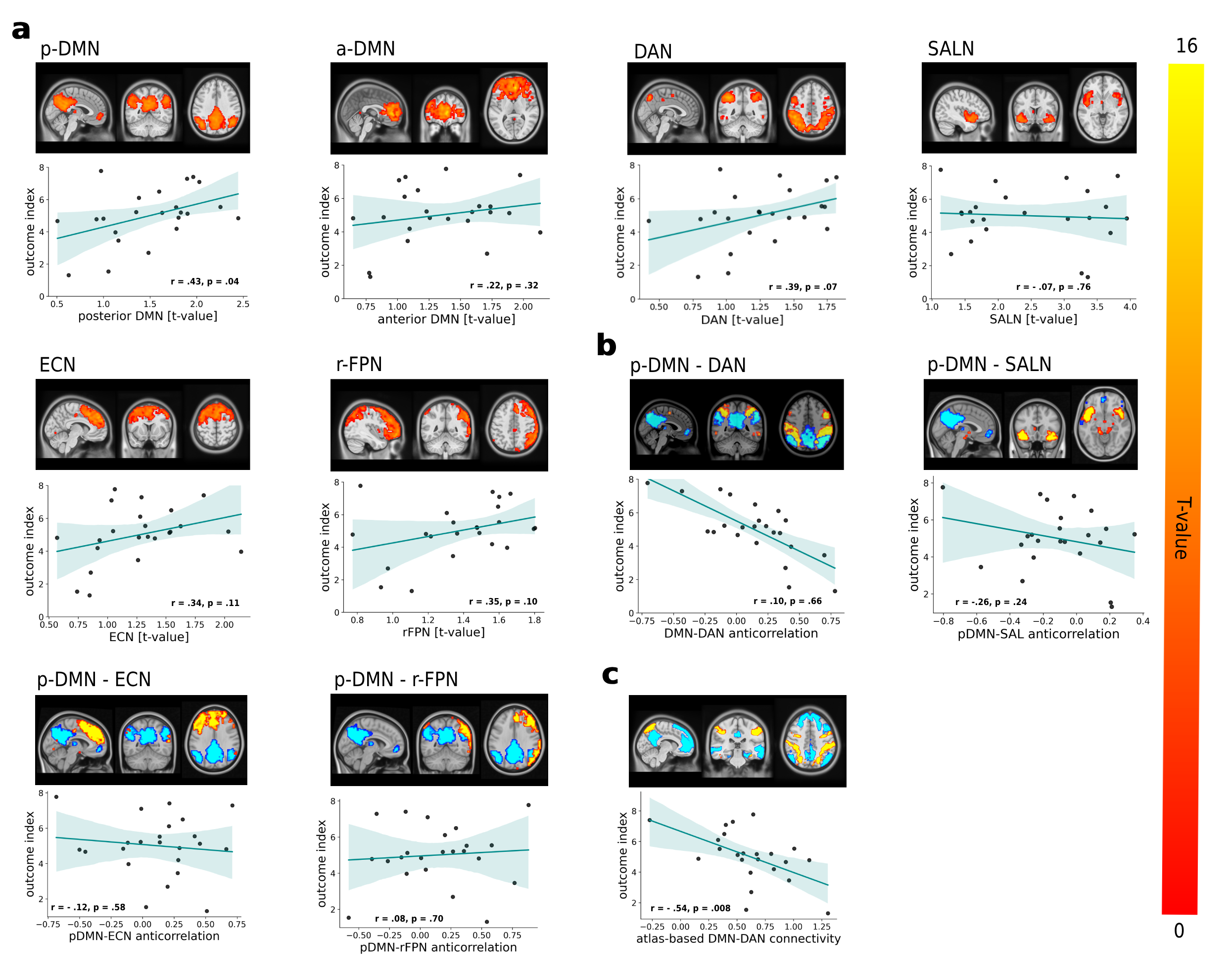
**Fig.S3 Average spatial maps of the independent components representing the default mode and executive functioning (EFN) related networks, and the atlas-based connectivity between the default mode and dorsal attention networks. (A)** *Upper*: Average spatial connectivity maps obtained with the group independent component analysis showing the posterior default mode network (p-DMN), anterior default mode network (a-DMN), dorsal attention network (DAN), salience network (SALN), executive control network (ECN), and the right fronto-parietal network (r-FPN). The color bar on the right represents the intensity (t-value) of the maps’ spatial connectivity. *Lower*: Scatter plots showing correlations between the spatial connectivity (extracted using an above-threshold mask) and the outcome index at the time of discharge for each resting state network. (B) Brain masks and scatter plots representing the correlation between the outcome index and the functional connectivity between the p-DMN and the a-DMN, DAN, SALN, ECN, and the r-FPN, respectively. **(C)** *Upper*: Brain masks representing the default mode network (DMN) in blue and the dorsal attention network (DAN) in yellow obtained from the Yeo 2011 [17] resting state networks atlas. *Lower*: Scatter plot showing correlations between the DMN-DAN connectivity and the outcome index. The shaded areas represent the 95% confidence interval of the fitted line.
